# From white dot syndromes to primary inflammatory choriocapillaropathies (PICCPs): updating and expanding the spectrum through multimodal imaging

**DOI:** 10.1186/s40942-026-00885-4

**Published:** 2026-06-23

**Authors:** Raul N. G. Vianna, Remo T. Moraes, Heloisa Nascimento, André Messias, Juliana Oréfice, Marcia Brazuna, Luciana F. Peixoto, Rodrigo Meirelles, Antônio Marcelo B. Casella

**Affiliations:** 1 Department of Ophthalmology, Fluminense Federal University (UFF), Niterói, RJ Brazil; 2 Brazilian Institute of Ophthalmology (IBOL), Rio de Janeiro, RJ Brazil; 3 Department of Ophthalmology, Federal University of São Paulo (UNIFESP), São Paulo, SP Brazil; 4 Department of Ophthalmology, State University of São Paulo, Ribeirão Preto, SP Brazil; 5 Centro Brasileiro de Ciencias Visuais (CBCV), Belo Horizonte, MG Brazil; 6 State University of Rio de Janeiro (UERJ), Rio de Janeiro, RJ Brazil; 7 Department of Ophthalmology, State University of Londrina (UEL), Londrina, PR Brazil; 8 Rua General Mariante, 88/802, Rio de Jane, RJ 22221-100 Brazil

**Keywords:** Multiple evanescent white dot syndrome, Acute posterior multifocal placoid pigment epitheliopathy, Idiopathic multifocal choroiditis, Relentless placoid chorioretinitis, Persistent placoid maculopathy, Serpiginous choroiditis, PICCPs, Primary inflammatory choriocapillaropathies, White dot syndromes, White spot syndromes

## Abstract

**Background:**

The term “white dot syndromes” (WDS) has historically been used as a descriptive label to encompass a heterogeneous group of inflammatory chorioretinal disorders characterized by multifocal whitish fundus lesions. Although clinically convenient, this classification relied predominantly on ophthalmoscopic appearance and fluorescein angiography findings and failed to identify the primary anatomical site of inflammation or the underlying pathogenic mechanisms. Consequently, profoundly disparate entities—such as acute posterior multifocal placoid pigment epitheliopathy (APMPPE), diffuse unilateral subacute neuroretinitis (DUSN), and HLA-A29 birdshot retinochoroiditis, among others—were inappropriately grouped together despite fundamentally different immunological substrates and target tissues.

**Methods:**

This narrative review revisits the historical evolution of the WDS concept and synthesizes evidence derived from multimodal retinal and choroidal imaging, including fundus autofluorescence, indocyanine green angiography, optical coherence tomography, and optical coherence tomography angiography, to identify shared pathogenic mechanisms and clinically relevant distinctions among them.

**Results:**

Advances in multimodal imaging over the past two decades have substantially reshaped the understanding of these disorders, demonstrating that a subset of conditions previously classified as WDS share a common pathogenic mechanism characterized by primary inflammatory ischemia of the choriocapillaris, with secondary involvement of the outer retina and retinal pigment epithelium. These findings support grouping these disorders under the concept of *primary inflammatory choriocapillaropathies* (PICCPs). Conventionally, this group includes APMPPE, idiopathic multifocal chorioretinitis, relentless placoid chorioretinitis, and serpiginous choroiditis (SC). Multiple evanescent white dot syndrome (MEWDS) is likewise included, although its pathophysiology remains debated, particularly regarding whether the primary target is the choriocapillaris or the photoreceptors. MEWDS is included in this review given the clinical and imaging overlap with classical PICCPs. Another rare entity that shares overlapping clinical and imaging features with SC—known as persistent placoid maculopathy (PPM)—may also be considered within this spectrum.

**Conclusion:**

This paper revisits the concept of PICCPs, which more accurately reflects the underlying pathophysiology of this group of disorders than the outdated WDS classification. Emphasis is placed on the role of multimodal imaging in refining disease classification, assessing disease activity and reversibility of ischemic injury, and guiding therapeutic decision-making. We also propose extending the PICCPs spectrum to include PPM. Adoption of a mechanism-based framework has important clinical implications for diagnosis, management, and prognosis.

## Introduction and historical perspective

In 1995, Benezra and Forrester proposed that the fundal white dots observed in several uveitic conditions likely represented a spectrum of a similar underlying pathological process rather than a collection of distinct and unrelated diseases [1]. At that time, disease classification was necessarily grounded in funduscopic appearance and fluorescein angiography (FA) findings, reflecting the technological limitations of the period. Within this context, the term white dot syndromes (WDS) emerged as a convenient descriptive umbrella encompassing a heterogeneous group of inflammatory chorioretinal disorders that shared overlapping ophthalmoscopic features [2–4].

Although conceptually practical, this appearance-based approach had inherent limitations. Grouping disorders solely based on similar fundus patterns did not allow accurate identification of the primary anatomical site of inflammation, nor did it adequately explain the marked heterogeneity observed in disease severity, recurrence rates, treatment response, and long-term visual outcomes. As a result, while the WDS terminology facilitated clinical communication, it lacked a robust pathophysiological foundation capable of guiding prognostication and individualized management [5, 6].

In the sustained pursuit of a more appropriate terminology for WDS, the term “white spot syndromes” has been proposed as a replacement; however, this change is largely semantic and does not overcome the fundamental conceptual limitations of the original nomenclature [7]. Similarly, the designation “non-infectious posterior uveitis” (NIPU) has been introduced as an alternative classification, but it remains imprecise. As a broad and ill-defined term, it lacks pathophysiological specificity and fails to encompass important entities such as sarcoidosis and Behçet disease. These conceptual inconsistencies underscore the persistent difficulty in defining a more appropriate and pathophysiologically meaningful terminology [8, 9].

Recently, in an effort to achieve greater diagnostic consensus for the most prevalent uveitic entities, the Standardization of Uveitis Nomenclature (SUN) Working Group established standardized classification criteria for these conditions through an international collaborative initiative involving experts in uveitis, ocular imaging, informatics, and machine learning [10]. Although most cases presented in this review were diagnosed before the SUN classification criteria, they fulfilled the currently accepted diagnostic criteria for their respective diseases.

The introduction and widespread adoption of fundus autofluorescence (FAF), indocyanine green angiography (ICGA), spectral-domain optical coherence tomography (SD-OCT), and optical coherence tomography angiography (OCT-A) represented a major turning point in the understanding of the posterior uveitides [11–13]. These imaging modalities enabled in vivo assessment of choroidal circulation and precise localization of inflammatory involvement within the chorioretinal complex. Importantly, they revealed that disorders previously grouped together on purely phenotypic grounds may differ substantially in their primary site of pathology [11–13].

While some conditions traditionally included among the WDS predominantly affected retina photoreceptors, such as acute zonal outer occult retinopathy (AZOOR), or the choroidal stroma, as HLA A29 birdshot retinochoroiditis, a distinct subset of disorders consistently demonstrates primary involvement of the choriocapillaris, with secondary changes affecting the outer retina and RPE [14, 15].

In 1972, during the pre-multimodal imaging era, Deutman et al. first proposed a primarily choriocapillaris-centered pathophysiological mechanism underlying acute posterior multifocal placoid pigment epitheliopathy (APMPPE) [16]. He suggested the term acute multifocal ischemic choriocapillaritis (AMIC), in contrast to Gass, who originally considered APMPPE to be a primary disease of the RPE [17].

In the early 2000s, with the systematic use of ICGA, the Lausanne group (Switzerland), led by Carl P Herbort Jr, recognized that APMPPE, idiopathic multifocal choroiditis (MFC), relentless placoid chorioretinitis (RPC), and serpiginous choroiditis (SC) share a predominantly vascular-centered pathophysiological mechanism involving inflammatory ischemia of the choriocapillaris [18, 19]. They therefore proposed the concept of *primary inflammatory choriocapillaropathies* (PICCPs) to encompass these entities [17, 18]. Multiple evanescent white dot syndrome (MEWDS) has also been included within this spectrum; however, its precise pathophysiology remains a matter of debate, particularly regarding whether the primary site of involvement is the choriocapillaris or the photoreceptors [20–23]. Despite this uncertainty, MEWDS is included in the present review due to its significant clinical and multimodal imaging overlap with classical PICCPs.

Based on these pathophysiological criteria, another rare form of placoid choriocapillaritis that shares overlapping clinical and imaging features with SC—known as persistent placoid maculopathy (PPM)—can also be encompassed within this group [24–26].

It is important to emphasize that, although PICCPs share inflammation and ischemia of the choriocapillaris as a central pathogenic event, they represent distinct clinical entities rather than a single disease continuum [27]. Accordingly, they differ in their natural history, extent and reversibility of ischemic injury, therapeutic requirements, and visual prognosis. Recognizing these distinctions is essential, as delayed or inadequate treatment in entities characterized by extensive or recurrent choriocapillaris ischemia may result in irreversible visual loss, whereas unnecessary immunosuppression in self-limited conditions may expose patients to avoidable risks without clear benefit [27].

The main clinical and demographic features of PICCPs are summarized in Table 1.

**Table 1:** Clinical and demographic features of MEWDS and classical PICCPs

Entity	Age	Sex	Laterality	Myopia	Viral prodrome	CNV	Treatment	Recurrence
MEWDS	20–40	Female	Usually unilateral	Frequent	Up to 50% of cases	Rare	Observation ± corticosteroids	Rare
APMPPE	20–40	None	Bilateral (often asymmetric)	Uncommon	Frequent	Rare	Observation ± corticosteroids	Rare
MFC	20–45	Female	Bilateral (often asymmetric)	Frequent	Occasional	30% of cases	Corticosteroids ± immunosuppression; anti-VEGF	Frequent
RPC	20–40	None	Bilateral, progressive	Uncommon	Occasional	Not reported	Corticosteroids ± immunosuppression	Frequent
PPM	>60	Male	Bilateral	Uncommon	25% of cases	40% of cases	Corticosteroids ± immunosuppression; anti-VEGF	None
SC	30–50	None	Bilateral, progressive	Uncommon	Rare	35% of cases	Corticosteroids ± immunosuppression	Frequent

Abbreviations: PICCPs = Primary inflammatory choriocapillaropathies; MEWDS = Multiple evanescent white dot syndrome; APMPPE = Acute Posterior Multifocal Placoid Pigment Epitheliopathy; MFC = Idiopathic multifocal choroiditis; RPC = Relentless placoid chorioretinitis; PPM = Persistent placoid maculopathy; SC = Serpiginous choroiditis; CNV = Choroidal neovascularization; ±=Associated or not, based on specialist’s discretion

## Pathophysiological link between choriocapillaris function and PICCPs

The outer retina represents a metabolic watershed zone that relies predominantly on the choriocapillaris for oxygen and nutrient delivery, rather than on the retinal circulation. Photoreceptors are highly metabolically active and therefore especially vulnerable to hypoxemia. In the setting of inflammatory choriocapillaris non-perfusion, oxygen deprivation may result in damage and loss of photoreceptor outer segments [28].

In contrast, RPE cells exhibit a substantially higher resistance to oxidative stress and hypoxic injury compared with most other retinal cell types [29]. Experimental studies in animal models demonstrated that, under hypoxic conditions, RPE cells undergo hypertrophy, increasing their intracellular storage of nutrients as a survival mechanism. This adaptive response may inadvertently limit metabolic support to photoreceptors, thereby exacerbating photoreceptor dysfunction and degeneration [29].

In this context, the advent of high-resolution choroidal imaging enabled the recognition of a distinct pattern of inflammatory choriocapillaris non-perfusion, referred to as choriocapillaritis, which underlies the spectrum of PICCPs [30]. These entities are considered primary because the inciting trigger remains unidentified, in contrast to secondary choriocapillaritis associated with well-defined etiologies such as syphilis or tuberculosis [27]. Nevertheless, a viral or post-viral immune-mediated trigger is strongly suspected, as a substantial proportion of patients report a preceding flu-like illness before the onset of ocular symptoms [31].

Accordingly, the resulting clinical phenotypes of PICCPs reflect the degree and distribution of choriocapillaris ischemia, which are largely determined by the caliber of the affected vessels, the spatial extent of non-perfusion, and the temporal profile of inflammation [27]. The spectrum therefore ranges from mild end-capillary involvement, as observed in MEWDS (at least in part), to more extensive disease affecting larger or pre-capillary vessels, such as APMPPE, MFC, RPC, PPM and SC [27]. In milder forms, ischemia selectively damages the photoreceptor outer segments while sparing the more resistant RPE, whereas more severe ischemia leads to irreversible atrophy involving the outer retina, RPE, and choriocapillaris, with potential progression to full-thickness choroidal atrophy [27].

## Infectious conditions mimicking PICCPs

**Fig. 1:**
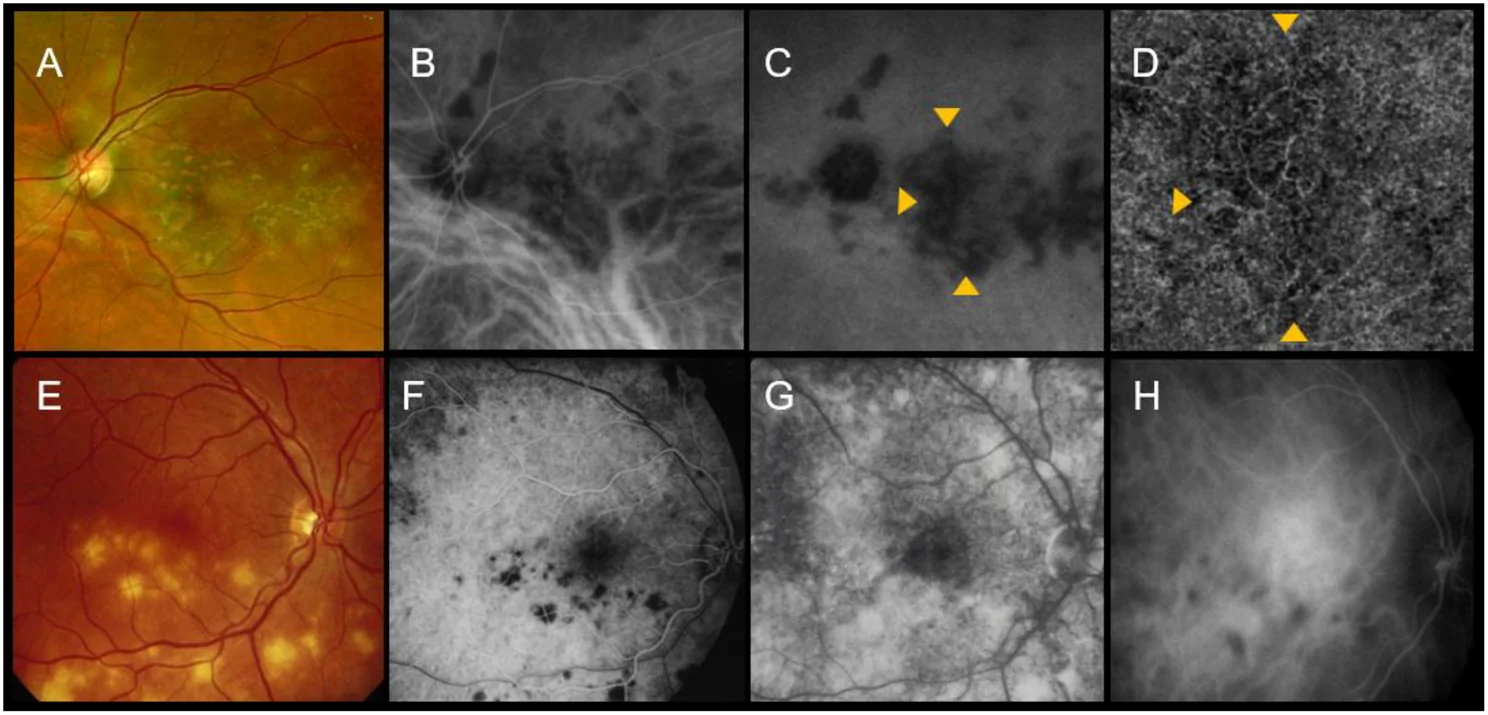
Multimodal imaging in serpiginous-like tuberculosis (**A-D**) and diffuse unilateral subacute neuroretinitis (DUSN) (**E-H**). (**A–D**) Serpiginous-like tuberculosis. (**A**) Color fundus photography shows yellowish placoid lesions involving the posterior pole. (**B, C**) ICGA reveals well-demarcated areas corresponding to choriocapillaris non-perfusion (arrowheads). (**D**) OCTA confirms flow deficits at the level of the choriocapillaris, consistent with inflammatory ischemia (arrowheads). (**E–H**) Diffuse unilateral subacute neuroretinitis (DUSN). (**E**) Color fundus photography shows multiple inferior yellow-white lesions. (**F, G**) FA demonstrates early hypofluorescence and late staining, respectively.(**G**) ICGA demonstrates hypofluorescent areas, indicating choroidal involvement

Before addressing the clinical and multimodal imaging features of PICCPs, it is important, particularly in developing countries, to emphasize the need to exclude degenerative disorders, as well as secondary inflammatory or infectious choriocapillaropathies. Conditions such as syphilis may produce placoid chorioretinitis or MEWDS-like presentations, while APMPPE-like lesions have also been described in association with syphilis, tuberculosis, diffuse unilateral subacute neuroretinitis-DUSN, bartonellosis, Zika virus infection, and cryptococcosis (Fig. 1) [32–41]. In these settings, the diagnosis of PICCPs frequently remains one of exclusion.

## Disease-specific entities

### MEWDS: primary choriocapillaritis or photoreceptorcentric disease (“photoreceptoritis”)?

**Fig. 2:**
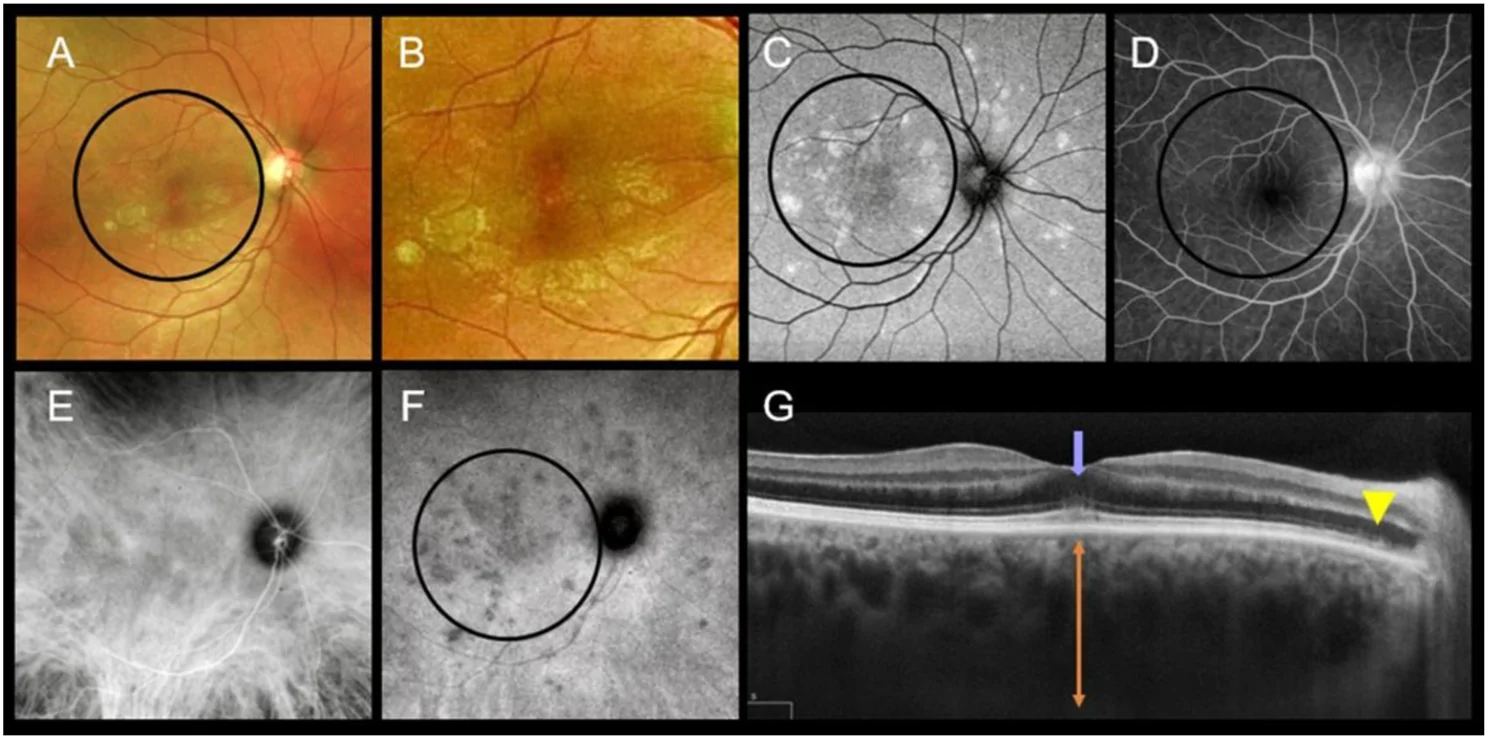
Multimodal imaging in MEWDS (**A-G**). (**A**) Color fundus photography shows multiple discrete yellow-white dots involving the posterior pole (black circle). (**B**) Magnified view highlights the characteristic granular aspect of the macula in detail. (**C**) FAF reveals hyperautofluorescent lesions. (**D**) FA demonstrates minimal changes in the posterior pole (black circle), with optic disc hyperfluorescence. (**E, F**) ICGA shows subtle abnormalities in the early phase and hypofluorescent lesions in the late phase (black circle). (**G**) SD-OCT shows disruption of the elipsoid zone (arrowhead), hyperreflective vertical lines extending from EZ/IZ towards inner limiting membrane, likely related to Müller cells activation (blue arrow) and a thickened choroid (orange double-headed arrow)

MEWDS was first described in 1984, independently and near simultaneously by Jampol et al. in the United States and by Takeda et al. in Japan [31, 42]. It typically affects young myopic women, presenting with acute unilateral visual loss, scotoma, photopsias, floaters and temporal visual field defects [31]. Half of patients refer to a flu-like episode before ocular symptoms, suggesting that infectious or post-infectious immune-mediated mechanisms may act as potential triggers in genetically or immunologically susceptible individuals. Fundus examination reveals mild vitreous inflammation, multiple yellow-white lesions at the level of the RPE or deep retina—predominantly in the parafoveal and peripapillary regions—foveal granularity, and mild optic disc involvement (Fig. 2A, B) [31]. Because of the transient nature of white dots, a characteristic finding is a yellow macula with granular aspect which can last longer and can be the only finding at the time of examination. Although MEWDS was originally included within the spectrum of PICCPs, its pathophysiology remains a matter of debate [20]. Indeed, ICGA and OCT-A findings differ substantially from those observed in classic PICCPs [7]. In the early phase of ICGA, MEWDS may show either no detectable abnormalities or only subtle changes. This contrasts sharply with classic PICCPs, in which prominent hypofluorescent lesions are typically observed in early ICGA frames due to choriocapillaris non-perfusion [18, 19]. In MEWDS, however, hypofluorescent lesions become more apparent during the mid-to-long phases, spreading centrifugally from the posterior pole, persisting into the very late phases (Fig. 2E, F) [43–45]. Some authors interpret these findings as representing areas of non-perfusion of small choriocapillaris vessels, thereby supporting the concept of primary choriocapillaris involvement [18, 19, 43, 44]. Others argue that these hypofluorescent areas instead reflect reduced uptake of indocyanine green by metabolically impaired RPE cells, thus favoring the hypothesis of primary involvement of the photoreceptor/RPE complex (“photoreceptoritis”) [20, 46].

OCT-A findings in MEWDS remain controversial. Only one report, describing two cases of acute MEWDS, demonstrated reduced choriocapillaris flow on OCT-A [47]. In contrast, Yannuzzi et al. failed to identify choriocapillaris flow deficits in two patients with acute MEWDS [48]. These findings lend support to the concept of primary involvement of the photoreceptor/RPE complex. However, proponents of the primary choriocapillaris hypothesis argue that current OCT-A technology may lack sufficient resolution to detect such subtle choriocapillaris flow abnormalities [43]. Rather than being mutually exclusive, these hypotheses may reflect different aspects or stages of the same disease process. Other multimodal imaging modalities further support the diagnosis of MEWDS. FAF typically demonstrates multiple punctate hyperautofluorescent lesions (Fig. 2C) [49]. FA shows multiple patchy hyperfluorescent lesions with a characteristic wreath-like appearance, which are more clearly visible in mid-to-late phases (Fig. 2D) [43–45]. SD-OCT shows a transient disruption of the ellipsoid zone or the interdigitation zone (Fig. 2E) [49, 50].

A MEWDS-like reaction, or secondary MEWDS (secMEWDS), has been increasingly reported in association with a wide range of underlying conditions [51]. These include non-infectious disorders such as MFC, infectious diseases such as tuberculosis and toxoplasmosis, as well as macular dystrophies, neoplastic conditions (primary vitreoretinal lymphoma, cancer-associated retinopathy), and immune checkpoint inhibitors drugs for the treatment of metastatic choroidal melanoma [51–53]. Current research suggests that secMEWDS is immunologically driven. Antigen exposure, triggered by viral infections, inflammation of choroidal vessels and Bruch’s membrane, traumatic subretinal hemorrhage, or choroidal rupture, can provoke this reaction [51]. Theoretically, secMEWDS can occur as an epiphenomenom in any disease affecting the outer retinal layers [51–53]. This results in an imaging pattern indistinguishable from primary MEWDS, underscoring the importance of identifying secMEWDS as a reactive manifestation that warrants investigation of an underlying cause [51]. Syphilis is not considered a cause of secMEWDS but rather a MEWDS-masquerader [35]. Although it may mimic typical multimodal imaging features, its pathophysiology might be related to direct *Treponema pallidum* infection rather than an immune-mediated process [35].

MEWDS is classically a self-limited condition with excellent visual prognosis [31, 42]. In most patients, observation and reassurance are sufficient, as spontaneous recovery usually occurs within weeks. Multimodal imaging demonstrates transient choriocapillaris hypoperfusion and/or “photoreceptoritis” with subsequent restoration of outer retinal structure [43, 44]. Systemic treatment is generally not required; however, a short course of oral corticosteroids may be considered in selected cases with marked foveal involvement (e.g., macular edema) or severe functional symptoms [54]. Choroidal neovascularization is rare and, when present, is managed with intravitreal anti-vascular endothelial growth factor (VEGF) [55]. Imaging follow-up is useful to document lesion resolution and exclude alternative diagnoses.

### APMPPE: is it truly “benign”?

APMPPE was initially described by Gass in 1968 and later redefined by Deutman, who suggested that the primary pathogenic mechanism is inflammatory hypoperfusion or non-perfusion of the choriocapillaris [16, 17]. This concept was subsequently supported by ICGA and further strengthened by OCT-A [19, 26]. The disease typically affects young adults (20–40 years), is usually bilateral and monophasic, and may be preceded by a flu-like illness [56]. Visual symptoms vary in severity and may include blurred vision, scotomas, and photopsias, and anterior segment inflammation can occasionally be present [56].

**Fig. 3:**
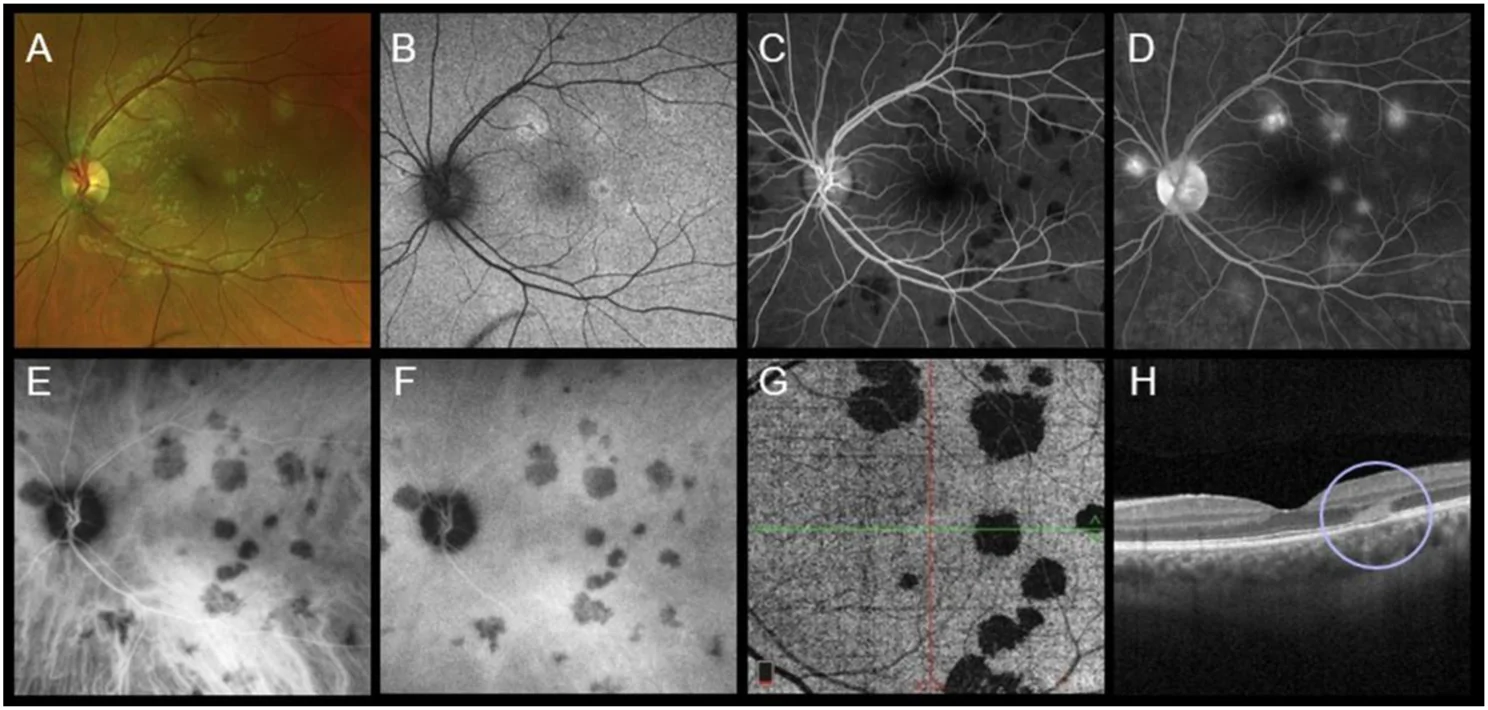
Multimodal imaging in APMPPE (**A-H**). (**A**) Color fundus photography shows multiple creamy-white lesions distributed across the posterior pole. (**B**) FAF reveals characteristic hyperautofluorescent lesions with central hypofluorescence. (**C, D**) FA demonstrates the classic pattern of early hypofluorescence followed by late hyperfluorescence. (**E, F**) ICGA shows persistent hypofluorescent areas throughout all phases, consistent with capillaris non-perfusion. (**G**) OCT-A confirms primary involvement of choriocapillaris, demonstrating corresponding flow voids. (**H**) SD-OCT shows hyperreflectivity of the outer retina, with involvement of the outer nuclear layer

Fundus examination reveals multifocal placoid yellowish-white lesions, at the level of the outer retina, with varying dimensions and located in the posterior pole and mid-periphery (Fig. 3A) [17]. FAF shows a mixed pattern, with hyperautofluorescence in areas of milder ischemia and hypoautofluorescence in regions of severe non-perfusion and RPE atrophy (Fig. 3B) [57]. FA demonstrates early hypofluorescence from non-perfusion followed by late pooling, supporting choriocapillaris ischemia as the primary pathogenic mechanism (Fig. 3C, D) [16, 17, 27]. ICGA is the gold standard for the evaluation of APMPPE, demonstrating scattered or confluent areas of early hypofluorescence that persist into late frames, corresponding to choriocapillaris non-perfusion (Fig. 3E, F) [58, 59]. OCT-A shows choriocapillaris dropout, correlating closely with ICGA hypofluorescent areas (Fig. 3G) [58–60].

SD-OCT findings vary according to disease stage, ranging from early outer retinal hyperreflectivity due to ischemic swelling to subsequent photoreceptor loss and outer retinal atrophy (Fig. 3H) [61]. Additional findings may include bacillary retinal detachment (BALAD), the recent described angular sign of Henle fiber layer hyperreflectivity (ASHH), and diffuse choroidal thickening [62, 63].

Although classically described as a self-limited and benign condition, approximately half of patients continue to complain of scotomas and metamorphopsia after resolution of the lesions [56]. Potential complications include chorioretinal scarring, secondary choroidal neovascularization, and rare but serious systemic associations such as cerebral or systemic vasculitis [64].

Therapeutic management of APMPPE depends on the extent of choriocapillaris ischemia, macular involvement, and disease course. Mild cases may be managed conservatively; however, local (sub-Tenon) or systemic corticosteroids are frequently considered when central vision is threatened or visual loss is significant [65, 66].

### MFC: punctate inner choroiditis (PIC) and multifocal choroiditis with panuveitis represent a single disease spectrum

**Fig. 4:**
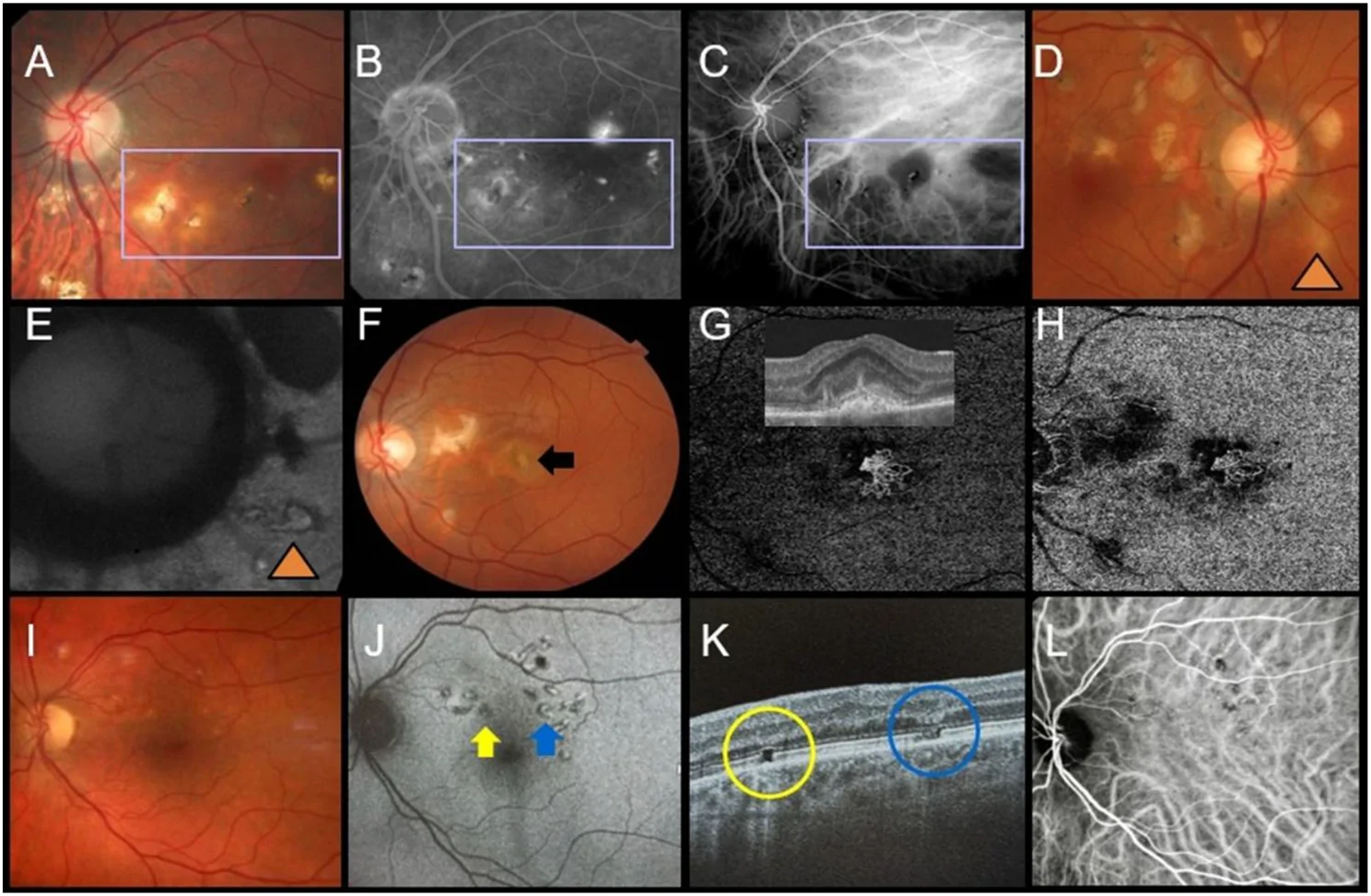
Multimodal imaging in MFC (**A-L**). (**A**) Color fundus photography shows multiple punched-out atrophic lesions involving the posterior pole (blue rectangle) and extending beyond the vascular arcades. (**B**) FA demonstrates a mixed pattern of hyperfluorescent and hypofluorescent lesions corresponding to those seen on color photography, reflecting different stages of lesion evolution due to recurrent activity (blue rectangle). (**C**) ICGA reveals hypofluorescent lesions that are more extensive than those observed on FA, indicating broader choriocapillaris involvement (blue rectangle). (**D**) Color fundus photography of an MFC case showing a new active lesion located inferonasal to the optic disc (orange arrowhead). (**E**) FAF demonstrates focal hyperautofluorescence at the site of the lesion (orange arrowhead), consistent with active inflammation and confirming disease recurrence. (**F**) Color fundus photography showing a greyish, round lesion with a creamy center, suggestive of inflammatory choroidal neovascularization (black arrowhead). (**G**) OCT-A delineates a type 2 neovascular membrane in the outer retina; Corresponding SD-OCT (detail) shows the characteristic inflammatory choroidal neovascular membrane with the “pitchfork sign.” (**H**) OCT-A at the level of the choriocapillaris demonstrates both the neovascular membrane and adjacent flow voids, consistent with underlying choriocapillaris involvement.(**I**) Color fundus photography of an acute MFC case showing small yellowish lesions in the posterior pole. (**J**) FAF reveals hypoautofluorescent lesions corresponding to chronic changes (yellow arrow) and a hyperautofluorescent lesion corresponding to active disease (blue arrow). (**K**) SD-OCT demonstrates these two lesions at different stages of evolution: the lesion marked by the yellow circle shows RPE atrophy, whereas the acute lesion shows ellipsoid zone disruption (blue circle). (**L**) ICGA shows hypofluorescent lesions corresponding to choriocapillaris non-perfusion

MFC is a unifying diagnostic entity encompassing disorders previously considered distinct, including multifocal choroiditis with panuveitis, PIC, and pseudo–presumed ocular histoplasmosis syndrome, following the 2013 consensus nomenclature [67–70]. Panuveitis is not a defining feature and is rarely observed [71]. MFC is characterized by recurrent, persistent inflammatory activity, presenting as creamy yellowish lesions that are typically bilateral and sequential, ultimately leading to permanent chorioretinal scarring (Figs. 4A, D, E and I) [67–70]. The disease predominantly affects young to middle-aged myopic women and presents with photopsias, visual field defects, and decreased vision when the macula is involved [71]. Some patients may present with chorioretinal linear streaks, predominantly located at the equator or around the optic disc, a presentation referred to as the *streaky MFC* phenotype [72]. These affected eyes are prone to developing choroidal neovascularization [72]. In recent years, additional phenotypic variants of MFC have also been recognized. One distinct subtype, termed *punctate inner pachychoroidopathy*, has been described in younger patients with emmetropia or low myopia and is characterized by thick choroids and a high prevalence of focal choroidal excavation [73]. Another noteworthy presentation is the so-called *“chrysanthemum-like phenotype”*, in which the chorioretinal lesions are surrounded by smaller satellite dots, creating a flower-like appearance [74]. While classical PIC lesions are generally confined to the posterior pole, chrysanthemum-like lesions frequently involve or exclusively affect the mid-peripheral and far peripheral retina [74]. *Solitary punctate chorioretinitis*, thus far reported in Chinese patients, is a rare subtype of MFC, in which a solitary macular lesion may underlie some cases of focal choroidal excavation and idiopathic choroidal neovascularization [75].

Rare reports describe sequential or overlapping occurrence of MFC and MEWDS in the same patient, suggesting partially shared inflammatory mechanisms involving the choriocapillaris and outer retina [76]. Multimodal imaging is essential for both diagnosis and follow-up. FA demonstrates window defects but is limited in detecting occult active lesions, whereas ICGA allows identification of both inactive scars and active areas of choriocapillaris non-perfusion (Fig. 4B, C, L) [77]. FAF typically reveals diffuse hyperautofluorescent lesions in the peripapillary region and posterior pole during the active phase, which subsequently evolve into hypoautofluorescent atrophic areas as inflammation subsides (Fig. 4E, J) [77].

SD-OCT findings typically include focal RPE elevation/disruption with associated hypertransmission, outer retinal hyperreflective material and ellipsoid zone loss, often accompanied by focal choroidal thickening [77]. A new OCT sign, termed “inflammatory plumes” has recently been described in MCF and consists of hyperreflective periatrophic infiltrates located at the borders of chorioretinal atrophy, representing persistent low-grade inflammation associated with progressive atrophic expansion [78]. OCT-A confirms the choriocapillaris involvement and is also valuable for detecting inflammatory choroidal neovascularization (Fig. 4F– H) [77].

In contrast to MEWDS and APMPPE, MFC does not resolve spontaneously. It follows a recurrent course and carries a significantly higher risk of inflammatory choroidal neovascularization, reported in up to 30% of cases [77]. Accordingly, early and aggressive systemic immunosuppressive therapy is recommended, with intravitreal anti-VEGF agents indicated for secondary choroidal neovascularization [77]. Macular edema may also occur and can be effectively managed with posterior sub-Tenon corticosteroid injections, alongside appropriate adjustment of systemic anti-inflammatory therapy [77, 79]. Close multimodal imaging follow-up is essential to detect both inflammatory reactivation and neovascular complications [77].

### RPC: immunosuppression as first-line therapy

RPC was first described by Jones et al. in 2000 and, due to its overlapping clinical features with APMPPE and serpiginous SC, has also been referred to as *ampiginous* choroiditis [80, 81]. RPC represents a rare and severe form within the spectrum PICCPs, characterized by a chronic, relapsing, and progressive course [81, 82].

**Fig. 5:**
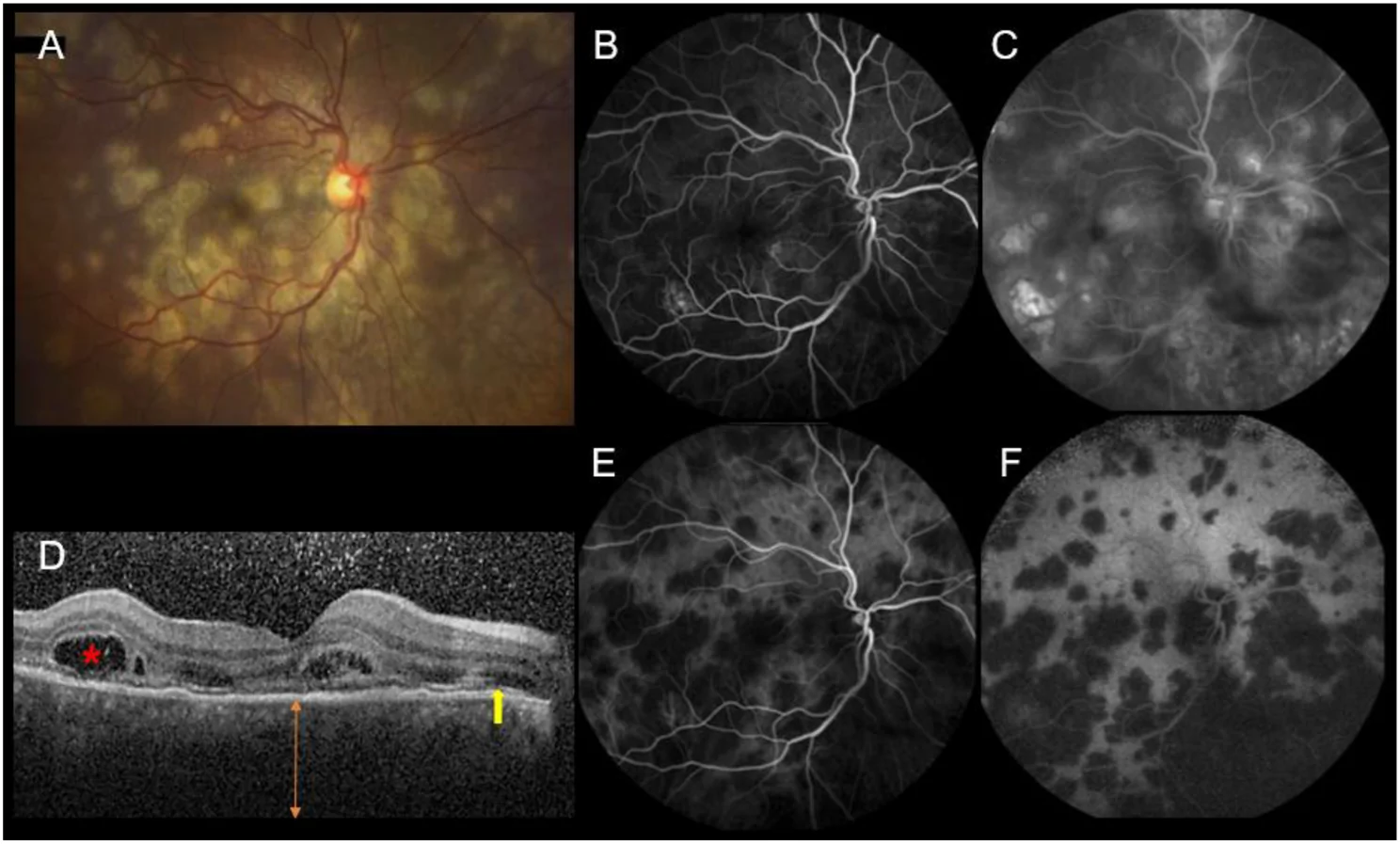
Multimodal imaging in RPC (**A-F**). (**A**) Color fundus photography shows multiple creamy-yellowish lesions distributed throughout the retina, extending beyond the midperiphery. (**B, C**) FA demonstrates a characteristic pattern of early hypofluorescence with subsequent hyperfluorescence. (**D**) SD-OCT reveals disruption of the ellipsoid zone (yellow arrow), bacillary layer detachment (red asterisc), and increased choroidal thickness (orange double- headed arrow) consistent with severe outer retina involvement. (**E, F**) ICGA shows persistent hypofluorecent lesions in all phases, confirming choriocapillaris involvement

It typically affects young to middle-aged adults, who present with decreased vision, scotomas, and photopsias [81, 82]. RPC is characterized by numerous, widely scattered chorioretinal lesions involving both the posterior pole and the mid-to-far peripheral retina, with the simultaneous presence of lesions at different stages of evolution, including active creamy placoid lesions and healing pigmented scars (Fig. 5A) [80–82].

Diagnosis relies on multimodal imaging. FAF demonstrates hyperautofluorescence in active lesions, which subsequently evolve into hypoautofluorescent areas as lesions heal. FA typically shows early hypofluorescence followed by late diffuse hyperfluorescence with ill-defined borders, corresponding to a “block early, stain late” pattern (Fig. 5B, C) [82, 83].

Similar to APMPPE and SC, ICGA reveals persistent hypofluorescence throughout all angiographic phases, consistent with choriocapillaris non-perfusion (Fig. 5E, F) [82]. Klufas et al. demonstrated using OCT-A that the choriocapillaris is the primary site of disease involvement [26].

On SD-OCT, acute changes predominantly affect the outer retinal layers, including disruption or loss of the ellipsoid zone, BALAD, focal hyperreflectivity at the level of the ellipsoid zone, or diffuse hyperreflectivity of the outer retina (Fig. 5D) [82].

RPC typically requires early, aggressive, and prolonged systemic immunosuppression, often incorporating steroid-sparing agents and, in refractory cases, biologic therapies [82, 84].

### PPM: there is a rationale for its inclusion within the PICCPs spectrum

**Fig. 6:**
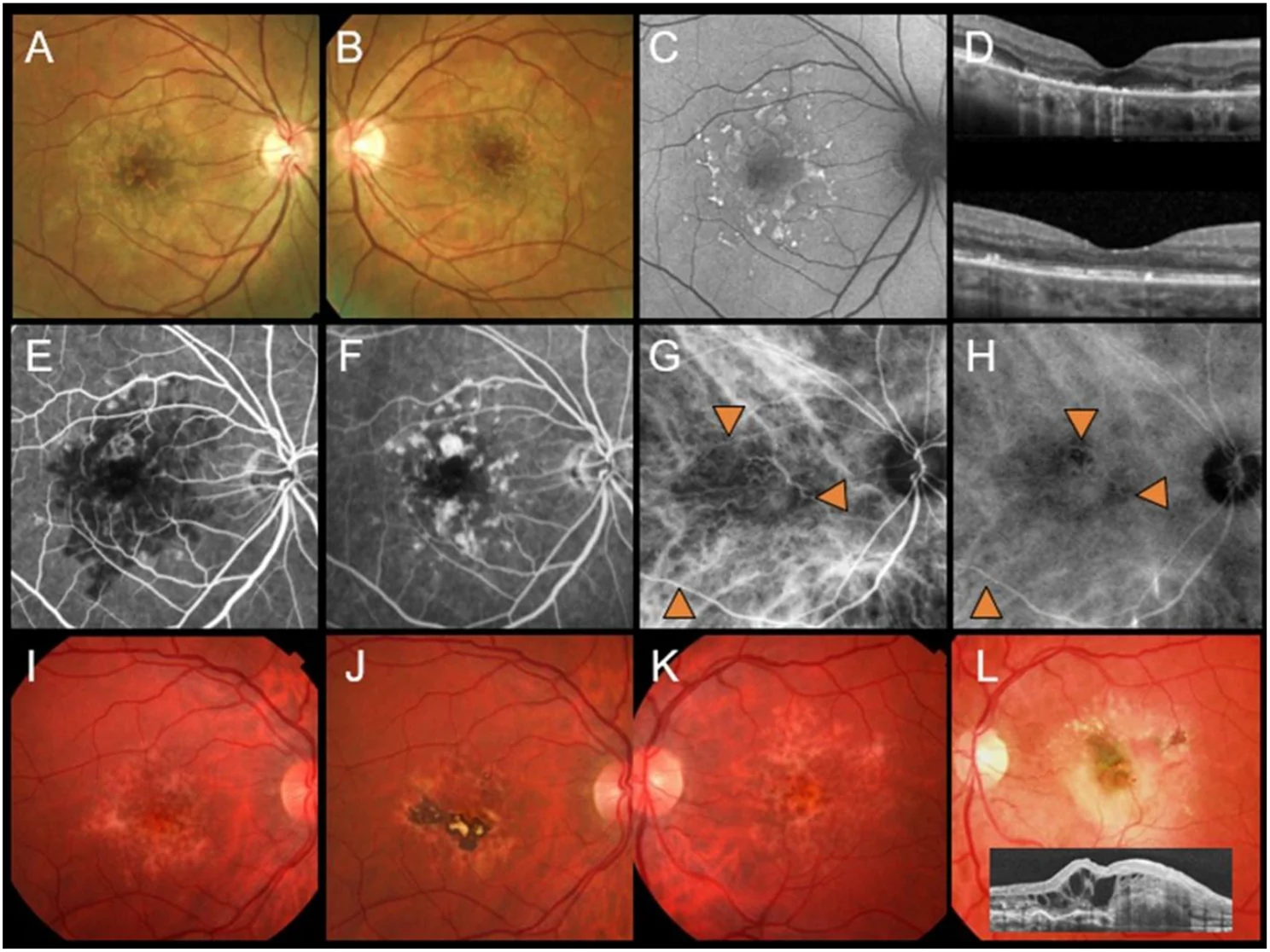
Multimodal imaging in PPM (**A-L**). (**A,B**) Color fundus photographs showing bilateral placoid, creamy-yellowish lesion involving the macula. (**C**) FAF demonstrates hyperautofluorescence corresponding to the acute phase of the disease. (**D**) SD-OCT at two time points: superior image (acute phase), shows disruption of the ellipsoid zone, hyperreflectivity of the outer nuclear layer, and hyperreflective choroidal dots, consistent with an inflammatory process. Best-corrected visual acuity (BCVA) was 20/50. Inferior image (2-year follow-up) demonstrates partial recovery of the central ellipsoid zone and resolution of hyperfluorescence changes in both the outer retina and choroidal. BCVA improved to 20/25. (**E, F**) FA shows early hypofluorescence of the placoid lesion with late hyperfluorescence, likely reflecting RPE alteration/atrophy. (**G, H**) ICGA reveals persistent placoid hypofluorescence throughout all phases of the examination (orange arrowheads), indicating sustained choriocapillaris non-perfusion. (**I, J**) Evolution of a PPM case from baseline to 1- year follow-up, demonstrating pigment migration within the placoid lesion. (**K, L**) Evolution of the left eye in the same patient, showing development of choroidal neovascularization. SD-OCT, in detail, demonstrates intraretinal cystic spaces and subretinal hyperreflective material located between the neurosensory retina and the RPE, highly suggestive of active choroidal neovascular membrane

PPM was first described by Golchet et al. in 2006 [24]. It represents a chronic and vision-threatening placoid disorder that predominantly affects older adults, typically in the sixth to seventh decade of life [24, 85]. The disease is characterized by bilateral, large placoid lesions primarily involving the macula and posterior pole, with incomplete lesion resolution and prolonged inflammatory activity (Fig. 6A, B, I-K) [85]. PPM appears to affect men more frequently [85]. Approximately 25% of patients report a preceding flu-like illness prior to the onset of ocular symptoms [85].

Clinically, patients typically present with mild to moderate visual loss, central scotomas, and metamorphopsia, reflecting sustained macular involvement [24, 85–87].

Diagnosis relies on multimodal imaging. FAF shows initial hyperautofluorescence of the lesions, which progressively evolves into hypofluorescence over time (Fig. 6C) [88]. On FA the lesions remain hypofluorescent till late and show minimal fluorescence in the late frames (Figs. 6E, F) [88]. ICGA demonstrates large, confluent areas of persistent choriocapillaris non-perfusion (Figs. 6G, H) [88]. SD-OCT reveals outer retinal hyperreflectivity, and disruption of external limiting membrane and ellipsoid zone (Fig. 6D) [88].

Importantly, OCT-A findings consistently indicate that the choriocapillaris is the primary site of disease involvement, providing strong pathophysiological support for its inclusion within the spectrum of PICCPs [26].

Choroidal neovascularization occurs in approximately 40% of affected eyes and represents a major determinant of poor visual outcomes (Fig. 6L) [85]. In such cases, intravitreal anti-VEGF therapy is indicated [89]. Notably, despite foveal involvement and persistent lesions, visual acuity often remains relatively preserved until the development of choroidal neovascularization [85, 90].

SC, particularly in its macular presentation, represents the most critical differential diagnosis of PPM. While macular SC exhibits centrifugal progression with active advancing borders, PPM follows a non-migratory, chronic course characterized by stable choriocapillaris non-perfusion [91, 92].

Given its chronic course and high risk of irreversible visual loss, early initiation and sustained use of systemic corticosteroids and/or immunosuppressive therapy are essential to control disease activity and limit progression [85, 87].

### Serpiginous choroiditis: the mandatory exclusion of tuberculosis

**Fig. 7:**
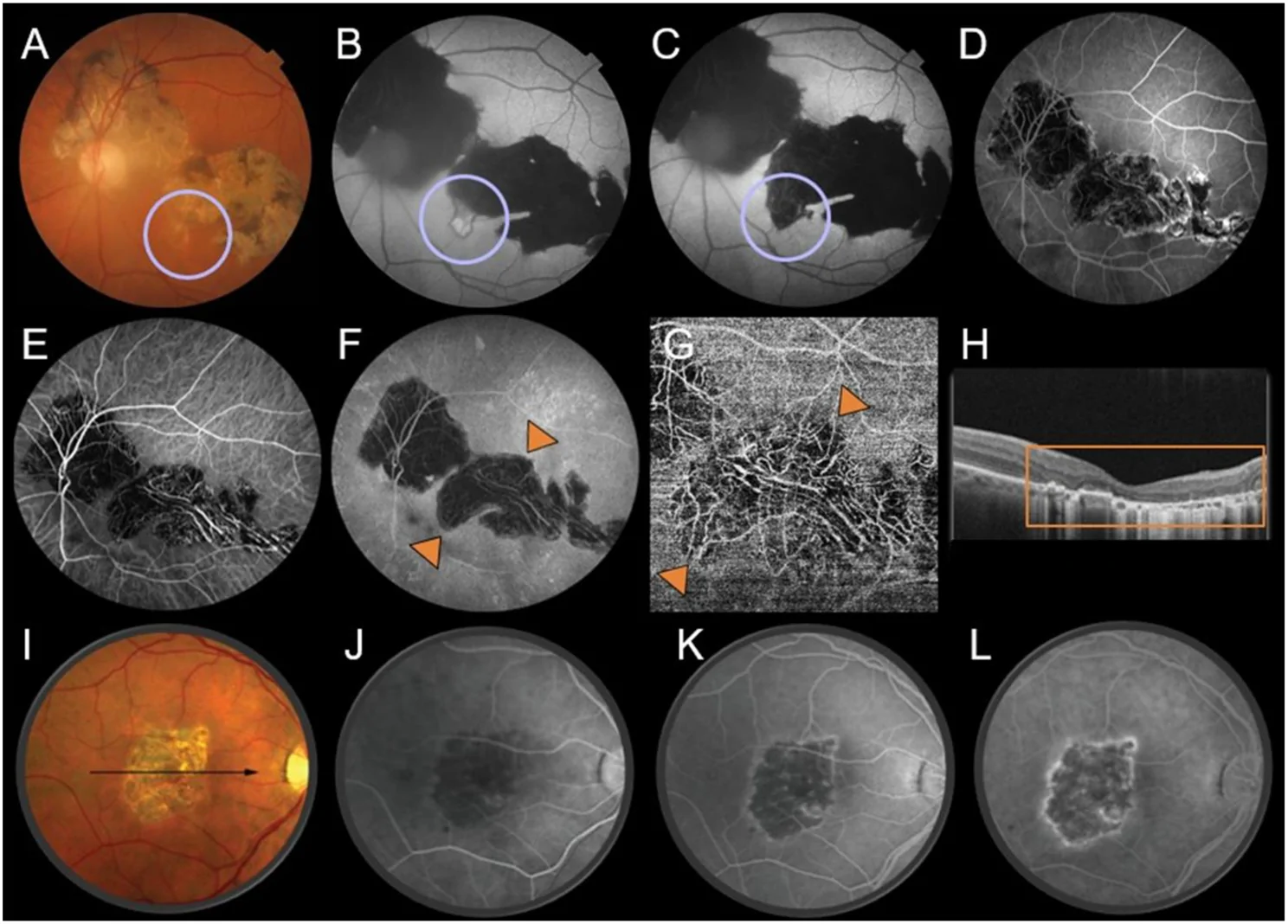
Multimodal imaging in SC (**A-L**). (**A**) Color fundus photography shows an atrophic lesion with a serpiginous configuration involving the border of the optic disc and extending into the posterior pole. Inferior to the macula, a creamy-yellowish lesion is observed (blue circle). (**B, C**) FAF images obtained at two time points demonstrate hyperautofluorescence of the lesion seen on color photography, indicating inflammatory activity (blue circle). Followig treatment with oral corticosteroids and azathioprine, the lesion became hypoautofluorescent (blue circle). (**D**) FA shows hypofluorescence of the central lesion with progressive hyperfluorescence of the borders. (**E, F**) ICGA reveals persistent hypofluorescent areas throughout all phases, consistent with choriocapillaris non-perfusion (orange arrowheads). (**G**) OCT-A confirms choriocapillaris involvement, revealing flow deficits that closely correspond to the hypofluorescent areas identified on ICGA (orange arrowheads). (**H**) SD-OCT demonstrates severe involvement of the outer retina, with associated RPE atrophy (orange rectangle). (**I-L**) (**I**) Color photography of an atrophic macular SC showing a placoid lesion involving the macula with sparing of the optic disc. (**J, K, L**) FA reveals a hypofluorescent lesion core associated with progressive hyperfluorescence at the lesion borders

The entity was first reported in 1932 by Paul Junius, who named it “retinochoroiditis parapapillaris” [93]. The term “serpiginous choroiditis” was later introduced by Laatikainen in the 1970s and subsequentially refined and definitively established by Gass in 1987 [94–96]. SC represents the most severe form within the PICCPs spectrum. It typically affects adults between 30 and 50 years of age, with no clear gender predilection [97]. SC is a bilateral, often asymmetric, progressive choriocapillaritis involving larger choriocapillaris vessels and is characterized by relentless centrifugal extension if not treated promptly and aggressively [97]. Clinically, patients present with vision loss, photopsias, and scotomas, usually with minimal anterior segment inflammation and mild vitritis [95, 96]. Funduscopic examination reveals characteristic yellow-white serpentine lesions that most commonly originate in the peripapillary region and initially spare the fovea (Fig. 7A) [96, 97]. Less frequently, lesion may arise from the central retina, the so called “macular SC” (Fig. 7I– L) [91, 92]. At presentation, tuberculosis-associated serpiginous-like choroiditis must be excluded using interferon-gamma release assay testing, as anti-tuberculous therapy is required in addition to immunosuppression in positive cases [98, 99]. Although the diagnosis is largely clinical, multimodal imaging is essential for disease monitoring. FAF demonstrates hyperautofluorescent borders corresponding to active inflammation and hypoautofluorescent atrophic centers, aiding in distinguishing active from healed lesions (Fig. 7B, C) [27]. FA reveals early hypofluorescence with late staining of active lesions [27, 96]. In chronic stages, lesion borders tend to become hyperfluorecent, while central areas remain hypofluorescent (Fig. 7D) [27, 97].

ICGA further delineates areas of choriocapillaris non-perfusion, identifying both active and atrophic regions, and may reveal hyperfluorescent halos suggestive of lesion progression (Fig. 7E, F) [27]. OCT-A confirms choriocapillaris dropout and enables the detection of secondary inflammatory choroidal neovascularization (Fig. 7G) [26, 100].

SD-OCT shows outer retinal disruption at lesion margins and full-thickness chorioretinal atrophy in chronic lesions (Fig. 7H) [27].

Choroidal neovascular membranes may develop in up to 35% of patients with SC, significantly contributing to visual impairment [97]. In such cases, intravitreal anti-VEGF may be indicated [101]. Early and sustained systemic immunosuppression is required to control disease activity and limit progressive choriocapillaris non-perfusion [102–105]. Treatment typically combines local and systemic corticosteroids with steroid-sparing immunosuppressive agents [102–105]. Prognosis is poor in the absence of early and aggressive therapy, and biologic agents—particularly anti–TNF-α therapies—are sometimes necessary to halt disease progression when conventional immunosuppression proves insufficient [106].

A comparative summary of multimodal imaging findings across PICCPs is provided in Table 2.

**Table 2:** Multimodal imaging characteristics of MEWDS and classical PICCPs

DISEASE	FUNDUS	FAF	FA	ICGA	SD-OCT	OCT-A (Choriocapillaris involvement)
MEWDS	Small whitish dots; foveal granularity	Transitory hyperAF dots/spots	Mid phase hyperfluor wreath-like lesions	Multiple late hypofluor spots	Ellipsoid zone disruption	Usually absent
APMPPE	Large placoid yellow-white lesions	HyperAF placoid lesions; hypoAF center	Early hypofluor lesions, late staining	Hypofluor placoid lesions	Outer retinal and RPE disruption; Acute APMPPE: ASHH sign, BALAD	Present
MFC	Punched-out scars; active yellow lesions	HyperAF active lesions, hypoAF atrophic scars	Early hyperfluor, hypofluor atrophic lesions	Multiple hypofluor lesions	Focal outer retinal disruption	Present
REC	Widespread placoid creamy, and atrophic lesions	HyperAF lesions, hypoAF atrophic lesions	Early hypofluor lesions, late staining	Persistent hypofluor lesions	Outer retinal/RPE disruption, BALAD	Present
PPM	Macular placoid lesion	Macular hyperAF plaques	Early hypofluor lesions, discrete late staining	Persistent macular hypofluor plaques	Chronic outer retinal damage Serous and fibrovascular PED	Present
SC	Geographic serpiginous lesion	HyperAF active borders, hypoAF center	Early hypofluor lesions, late staining borders	Extensive hypofluor lesions	Extensive outer retinal and RPE atrophy	Present

Abbreviations: PICCPs = Primary inflammatory choriocapillaropathies; MEWDS = Multiple evanescent white dot syndrome; APMPPE = Acute posterior multifocal placoid pigment epitheliopathy; MFC = Idiopathic multifocal choroiditis; REC = Relentless placoid chorioretinitis; PPM = Persistent placoid maculopathy; SC = Serpiginous choroiditis; FAF = Fundus autofluorescence; HyperAF = Hyperautofluorescent; HypoAF = Hypoautofluorescent; FA = Fluorescein angiography; Hyperfluor = Hyperfluorescent; Hypofluor = Hypofluorescent; ICGA = Indocyanine green angiography; SD-OCT = Spectral-domain optical coherence tomography; OCT-A = Optical coherence tomography angiography. ASHH = Angular sign of Henle fiber layer. BALAD = Bacillary layer detachment. PED = Pigment epithelium detachment. RPE = Retinal pigment epithelium

## Future perspectives and conclusion

PICCPs embody a paradigm shift from descriptive funduscopic taxonomy toward a mechanism-based classification of disorders previously subsumed under the outdated label “white dot syndromes.” By integrating APMPPE, RPC, MFC, and SC—and potentially extending this spectrum to include PPM—PICCPs provide a unified pathophysiological framework centered on choriocapillaris inflammation and ischemia. The inclusion of MEWDS within this spectrum remains a matter of ongoing debate, reflecting persistent uncertainties regarding its primary site of involvement. Continued advances in multimodal imaging are expected to further refine this concept, enabling more precise phenotyping, improved prognostication, and increasingly individualized therapeutic strategies.

## Data Availability

No datasets were generated or analysed during the current study.
